# Design and Reproducibility of Food Propensity Questionnaire for Characterizing Intake of Pyrethroid and Organophosphate Insecticides in Adolescents

**DOI:** 10.3390/children13030320

**Published:** 2026-02-25

**Authors:** Marija Macan, Antonija Sulimanec, Jelena Kovačić, Irena Keser, Breige McNulty, Anne Nugent, Željka Babić, Martina Pavlić, Darja Sokolić, Patricia Tomac, Adrijana Košćec Bjelajac, Veda Marija Varnai

**Affiliations:** 1 Division of Occupational and Environmental Health, Institute for Medical Research and Occupational Health, 10000 Zagreb, Croatia; 2 Faculty of Food Technology and Biotechnology, University of Zagreb, 10000 Zagreb, Croatia; 3 UCD Institute of Food & Health, School of Agriculture and Food Science, University College Dublin, D04 C1P1 Dublin, Ireland; breige.mcnulty@ucd.ie (B.M.);; 4 Institute for Global Food Security, School of Biological Sciences, Queen’s University Belfast, BT7 1NN Belfast, Ireland; 5 Department for Nutrition and Data Management, Croatian Agency for Agriculture and Food, 31000 Osijek, Croatia; 6 Faculty of Medicine, University of Rijeka, 51000 Rijeka, Croatia

**Keywords:** adolescent boys, dietary habits, pesticide residues, fruits and vegetables, risk assessment, self-report questionnaire

## Abstract

**Highlights:**

**What are the main findings?**

- A developed food propensity questionnaire (FPQ) targeting pesticide-relevant foods in adolescents showed good overall reproducibility.
- Obtained results indicate inadequate vegetable intake in young adolescent boys.

**What are the implications of the main findings?**

- The proposed design of the food propensity questionnaire should be utilized in future studies evaluating dietary exposure to pyrethroids and organophosphate insecticides among sensitive population groups.
- More educational initiatives should be implemented to encourage higher fruit and vegetable consumption among children from a young age.

**Abstract:**

Background/Objectives: There is currently no food propensity questionnaire (FPQ) developed specifically to address pesticide intake in the adolescent population. Therefore, the objective of our study was to design a specific FPQ with emphasis on fruit and vegetable consumption and dietary exposure to pyrethroids (PYR) and organophosphate (OP) insecticides and to test its reproducibility. Methods: The FPQ was designed for the purpose of this study primarily by identifying high-risk foods according to the EFSA annual reports on pesticide residues in food. In total, 99 parents/guardians of 10–12-year-old boys completed the first FPQ during May to June 2022 and again during October 2022 to January 2023. Results: For the whole questionnaire, comprising 111 questions covering presumed major sources of pesticides in a diet, the median Cohen’s weighted kappa was 0.607 (interquartile range, IQR 0.526–0.678, total range 0.275–0.864). Furthermore, similar good overall reproducibility was noted when we focused only on the presumed food sources of PYR and OP pesticides (54 questions; median kappa 0.624, IQR 0.535–0.695, total range 0.275–0.864). Best reproducibility was noted for tomatoes (fresh, in season), lettuces (generic), and pork lard. Median estimated fruit intake for 459 adolescents based on the FPQ was 262 g/day (IQR 176–376 g/day), and vegetable intake was 123 g/day (IQR 74—190 g/day). Conclusions: Due to its good reproducibility, this FPQ, which estimates PYR and OP pesticide exposure, represents a valuable tool for future epidemiological studies and public health surveillance that focus on pesticide residue exposure in specific populations.

## 1. Introduction

Fruits and vegetables are crucial components of every meal due to their valuable content of essential microelements and bioactive compounds, making them a basis for healthy dietary recommendations. Diets rich in fruits and vegetables are widely recommended for their health-promoting and protective properties, particularly in the prevention of cardiovascular disease and certain types of cancer [1,2]. Recently, Schwingshackl et al. [3] found that an increased intake of fruits and vegetables is associated with a decreased risk of all-cause mortality. Additionally, they observed that optimal consumption of fruits and vegetables along with whole grains, nuts, and fish could lead to a 56% reduction in all-cause mortality. Thus, most dietary recommendations encourage adults to consume at least five portions of various fruits and vegetables per day, with half of these portions consisting of vegetables [4,5]. Nutrition (and malnutrition) also plays a pivotal role in health during sensitive developmental windows such as adolescence and can have a profound influence on long-term health outcomes in adulthood. Adolescence is characterized by rapid physical growth, reproductive maturation, and ongoing brain development accompanied by substantial neurobehavioral changes. Adolescents, therefore, have increased nutritional requirements to support their growth spurts, muscle development, hormonal shifts, and overall health and functioning. Adequate intake of fruits and vegetables (above 400 g/day) has been associated with more favourable micronutrient profiles [6].

Unfortunately, the intake of fruits and vegetables is an important route of exposure to multiple pesticide residues. Pesticides are extensively used in agriculture, including the production of fruits and vegetables, to control weeds and insect infestations, both in their natural form and as chemically synthesized products [7]. Since the 1980s, pyrethroids (PYR) have been widely used due to their greater effectiveness and lower toxicity compared to other major groups of insecticides, including organophosphates (OP) and carbamates [8]. Pyrethroids target sodium channels, leaving them open and causing sustained depolarization, reducing acetylcholinesterase activity and altering cytochrome P450 activity [9], while organophosphates irreversibly inhibit acetylcholinesterase (AChE), which leads to the accumulation of acetylcholine in synapses and neuromuscular junctions of the central and peripheral nervous system. Given these primarily neuroactive mechanisms, developmental exposure to PYR and OP, including at relatively low exposure levels, has been associated with adverse neurobehavioral functioning and neurodevelopmental outcomes [10,11,12]. Pyrethroids and OP have, however, also been implicated in having the potential to disrupt the endocrine system [13,14]. There is evidence that even low dietary exposure to pesticide residues may affect hormonal and reproductive health as well as change pubertal timing, despite the small number of studies looking at the relationship between exposure to PYR and OP and reproductive health [15,16,17,18].

This area thus remains an active field of research, for which a reliable dietary assessment is a crucial part of risk assessment. While consumption data are usually collected through 24-h recalls or a combination of these recalls with the Food Frequency Questionnaire (FFQ), surveys assessing exposure to target food components can also use the Food Propensity Questionnaire (FPQ) for data collection [19]. The FPQ is a qualitative FFQ used to estimate long-term food intake, capturing primarily food variation and frequency [20,21]. As it does not include questions on portion sizes, it can be used alongside 24-h recall. FPQ typically includes a list of specific individual foods or food groups, which are consumed frequently or may increase dietary intake of relevant food-borne hazards. Similarly to FFQs, respondents are asked to indicate how often they typically consume foods—daily, weekly, monthly, or yearly. Recently, the FPQ was used to evaluate vitamin D intake in Slovenian adolescents [22], supplement consumption in Polish adults [23], and consumption of seldom-eaten foods in young children in Germany [24]. In summary, FPQ is a convenient tool for dietary assessment, but there is currently no FPQ developed that specifically targets possible PYR and OP intake in adolescents, with proven good reproducibility.

To address this need, this study aimed to test the reproducibility of a qualitative FPQ utilized to assess dietary exposure to PYR and OP insecticides with emphasis on fruit and vegetable consumption. Furthermore, the intake of fruits and vegetables in adolescent boys was evaluated in relation to the existing food-based guidelines in Europe and worldwide.

## 2. Materials and Methods

### 2.1. Study Design and Participants

Data were gathered within the longitudinal cohort study (PyrOPECh-Health) conducted in the City of Zagreb, Croatia, and its surrounding area, as part of the nationally funded PyrOPECh project (“Exposure to pyrethroid and organophosphate insecticides in children—risk assessment for adverse effects on neuropsychological development and hormonal status”, funded by the Croatian Science Foundation; grant no. IP-2019-04-7193). Invitations were distributed via e-mail to 56 schools located in the city of Zagreb and Zagreb County, and approval of school principals was sought in accordance with the National Pedagogic Standards of the Republic of Croatia [25]. Principals of 41 schools approved conducting the study in their schools. However, two of these schools dropped out due to organizational issues, leaving the total number of 39 schools (70% response rate), in which all the boys attending the 5th grade(s) and their parents/guardians (*n* = 1629) were invited to join the study via written study information packs. All in all, 459 agreed to participate and filled out the FPQ (28% response rate). The FPQ was part of a comprehensive general questionnaire for parents/guardians, comprising questions about the household, parents’ and boys’ characteristics and health, and boys’ diet characteristics, completed by 459 participants. For the purpose of testing the FPQ’s reproducibility, a subgroup of 102 parents/guardians was asked to complete the FPQ twice. Questionnaires that were completed by the mother at baseline and the father at repeated questionnaire, or vice versa, were excluded from the FPQ reproducibility testing. There were three such cases; therefore, the total number of questionnaires used for the FPQ reproducibility testing was 99. The FPQ was answered first during late spring (T0, May to June 2022), and the repeated FPQ was answered during late autumn or early winter (T1, October 2022 to January 2023). Parents/guardians asked to participate in the assessment of FPQ reproducibility were recruited based on the temporal sequence in which schools were recruited (e.g., the first school where the survey was conducted was contacted for FPQ repeatability testing, then the second, third, etc.). The response rate of parents/guardians for the reproducibility of the FPQ was 76% (out of the 134 asked parents/guardians, 32 declined participation).

### 2.2. Dietary Assessment

#### 2.2.1. Food Propensity Questionnaire

The Food Propensity Questionnaire (FPQ) was developed in 2022 with the aim of assessing the dietary intake of pesticides and toxic metals for the cohort of adolescent boys in the PyrOPECh study. The FPQ comprised 111 questions. The first section included questions assessing general dietary habits, including the consumption of food categories that are commonly avoided for different reasons, such as animal-based foods (meat, fish, eggs, milk) and grains/cereals. Participants were also asked about any dietary restrictions or specific diets, and if so, which. The following two questions asked how often they consumed organic and home-grown foods. The main part of the FPQ consisted of a comprehensive food list for which parents/guardians reported how frequently, over the past 12 months, their son had consumed each item. Foods were categorized mainly based on the FoodEx2 categories, and other research papers [26,27,28], into nine groups: grains and grain-based products; vegetables and legumes (fresh, cooked, or seasoning); fruit and fruit-based products; meat and meat-based products; fish; milk, dairy products and milk substitutes; eggs; fats and oils; and sweets/sweeteners and beverages. The frequency was defined as never, less than once a month, 1–3 times per month, once a week, 2–3 times per week, 4–5 times per week, and 6–7 times per week. For certain food items (mostly vegetables and fruits), the frequency of consumption during their peak seasons compared to the off-season was requested (Table 1).

There were several phases of selection of food items in the FPQ, considering both previous measurements of these substances in food and the expected dietary intake in the Croatian male adolescent population (Supplementary Table S1). First, as described in our previous paper on the Total Diet Study (TDS) methodology from an earlier phase of the PyrOPECh project [29], high-risk foods with respect to pesticide burden were identified using the EFSA annual reports on pesticide residues in food (EU-coordinated control programmes, EUCP). These reports summarize measurements of the pesticide residues for the selected foods, mainly fruits and vegetables, in the European market across one year. The last five reports available at the time of questionnaire development were used, i.e., reports for the period 2015–2019 [30,31,32,33,34]. With this programme, there is an assurance of consistent data collection across the EU to assess compliance with maximum residue levels (MRLs). The EUCP is conducted annually with a diverse range of food items, as shown in Table 2. Three criteria proposed by Chiu et al. [35] were applied: (1) the proportion of samples with quantifiable residues; (2) the proportion of samples with residues above the maximum residue level; and (3) the proportion of samples with at least three different detectable pesticide residues. Foods that were in the top tercile according to any of the three criteria were considered high-risk foods. Finally, as described in detail in Sulimanec et al. [29], the expected food consumption in Croatian male adolescents was estimated based on the consumption data for Croatian adults and Slovenian adolescents from the EFSA Comprehensive European Food Consumption Database (https://www.efsa.europa.eu/en/data-report/food-consumption-data, accessed on 20 April 2021), as consumption data for Croatian adolescents was not yet available from this database at the time of questionnaire development. Lastly, in addition to the foods identified based on the described data sources, other foods were included based on the expert advice of nutritionists in the project team as presumed important dietary contributors to the chemical substances of interest or specific to the adolescent population.

Conversely, there were food items identified as important contributors to dietary pesticide burden based on the EFSA reports but not included in our FPQ: fungi, fresh figs, granate apples/pomegranates, and grapefruit. The latter three were omitted from the FPQ due to the expected low consumption in Croatian adolescents (up to 1% of consumption in the category of fruits for Croatian male adults; up to 0.5% for Slovenian male adolescents). Wild fungi were not included due to difficulties with obtaining representative food samples for pesticide residue measurements in the Croatian market, while cultivated fungi were omitted as they were in the bottom tercile according to all three criteria (only processed fungi were ranked in the top tercile). All highly consumed fruits and vegetables were included in the FPQ, except for onion (9% and 10% of consumption in the corresponding category for Slovenian adolescents and Croatian adults, respectively), as it was in the bottom tercile according to all three criteria for high-risk foods for pesticides.

As a last step in FPQ design, we grouped certain items based on the expert judgement of nutritionists in the project’s team and recommendations from previous studies with the purpose of avoiding a lengthy FPQ list [39]. Examples of food items grouped in the FPQ are apples and pears, apricots and peaches, and spinach and Swiss chard. Then, some food items in the FPQ list were divided into distinct categories based on processing state. For example, tomatoes and sweet peppers were each classified separately as fresh or thermally processed, as both foods are commonly consumed in raw and cooked forms. Food items with a high percentage of consumption based on available literature data for this region (nuts, potatoes, carrots, courgettes, and legumes) [26,27] were also included in the FPQ list, regardless of the EU-coordinated control programmes (EUCP) and national programme results.

#### 2.2.2. Identification of Contributors to PYR and OP Insecticides Dietary Exposure

For the purpose of assessing whether this FPQ covered foods in which pesticides (PYR and OP) were found during the period when the project’s first wave was conducted, we used data from EU-coordinated control programmes (EUCP) from 2021 to 2023. Every three years, food items are mostly repeated, allowing the trend of pesticide residues to be followed over time. This paper used results from 2021 to 2023, as those were the years when the study was conducted.

### 2.3. Estimation of Fruit and Vegetable Intake

Fruit and vegetable intake was estimated for 459 boys for which a general questionnaire, including the FPQ, was completed. To estimate the consumption of fruits and vegetables in grams per day using the FPQ, categories of consumption frequency were first expressed as the number of consuming days per year. For example, the FPQ category “4–5 times per week” was assigned a value of 234 consuming days per year, obtained as 52 (number of weeks in a year) multiplied by 4.5 (midpoint of the corresponding FPQ category). In cases when seasonal and off-seasonal consumption were assessed separately in the FPQ, consuming days during each period were calculated separately and then summed to obtain the total number of consuming days in a year.

Food consumption in grams per consuming day was approximated using the database from the Croatian national food consumption survey on adolescents and adults aged 10 to 99 years [28]. This database is part of the Comprehensive European Food Consumption Database, which is a repository of detailed information on the dietary habits of citizens across the EU compiled by EFSA. We used summary consumption data for 130 male teenagers, aged 13 to 19, who participated in the Croatian survey, expressed as edible portions of raw foods. The mean value of acute food consumption grams in a single consuming day was utilized for foods that are typically consumed raw and have at least ten consuming days in the database. Weight yield factors [40,41], which represent the weight change resulting from thermal processing, were used in the mean acute food intake values for typically thermally processed goods in order to determine the quantity of food consumed. The selected yield factors reflected the distribution of commonly applied thermal processing types in the Croatian population, identified from the database of the Croatian National Adult Consumption Survey [28]. The yield factors were applied to all vegetables except for lettuce, cucumbers, spring onions, melons, pickled/marinated vegetables, head cabbages, tomatoes, and peppers, which are most often eaten raw. Although it is not uncommon to thermally process head cabbages, tomatoes, and peppers in Croatian cuisine (in at least 20% of meals from the database of the Croatian National Adult Consumption Survey, these foods were thermally processed), the consumption of raw and thermally processed foods was not available as separate values for the adolescent population. Thus, for these three foods, the consumption values from the EFSA database were paired with frequency data expressed for the sum of raw and thermally processed foods.

When the number of consuming days per food item for Croatian male adolescents was less than 10, data for male adolescents from the surrounding countries sharing a similar cuisine (Bosnia and Herzegovina, Slovenia, and Serbia) available from EFSA’s database were used. In such cases, the weighted mean of all four countries was calculated. The weights were equal to the number of consumers for Bosnia and Herzegovina, Slovenia, and Serbia, while the weight for Croatian data was calculated as the number of consumers multiplied by 2, i.e., one Croatian participant had the same contribution as two consumers from the region. For fruit and vegetable intake calculation, missing FPQ data for foods consumed throughout the whole year and for seasonal consumption (up to 5% of missing entries per question) were entered by mean imputation. Missing data for off-season consumption were then imputed by simple regression imputation, where the only predictor was the seasonal consumption of the corresponding food. Fruit and vegetable intake was calculated separately by summing consumption for all food items from the respective category included in the FPQ. Fruit nectars, juices (except for freshly squeezed), and concentrates were excluded from the calculation, as well as potatoes and legumes. In addition to the FPQ, questions were also asked about dietary restrictions (meat, fish, eggs, milk, and grains) and consumption of organic or home-grown foods.

### 2.4. Data Analysis

Cohen’s kappa statistics with squared weights, with higher penalization of larger discrepancies between responses in the original questionnaire (T0) and those in the validation questionnaire (T1), were used to evaluate the reproducibility. Reproducibility was classified according to Landis and Koch’s criteria [42]. To test the difference between food consumption in season and out of season, the Wilcoxon matched-pairs test was used.

## 3. Results

**Figure 1:**
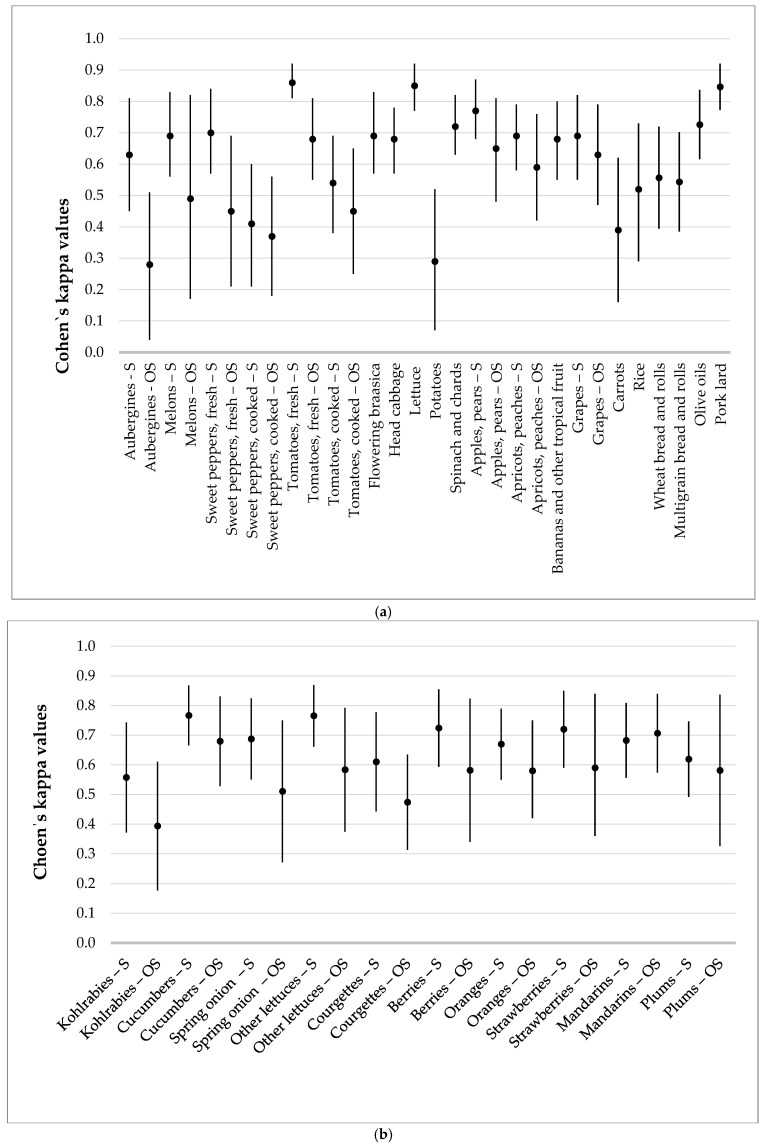
S—in season, OS—out of season (**a**) Cohen’s kappa values with 95% confidence interval for food products that had PYR and OP insecticide residues quantified in EU-coordinated control programmes (EUPIC) from 2021 to 2023. (**b**) Cohen’s kappa values with 95% confidence interval for fruits and vegetables that can be eaten in season and out of season.

For the whole questionnaire, 111 questions covering presumed major sources of pesticides in diet, the median Cohen’s weighted kappa was 0.607, with an interquartile range of [0.526–0.678] and a total range of [0.275–0.864]. Furthermore, similar good overall reproducibility according to Landis and Koch’s criteria was noted when we focused only on the presumed food sources of PYR and OP pesticides, as well as the seasonality of fruit and vegetable consumption. Specifically, the kappa value for these 54 questions was 0.624, with an interquartile range of [0.535–0.695] and total range of [0.275–0.864]. The Cohen’s weighted kappa values and their interquartile ranges for each question are presented in Figure 1a,b and in Supplementary Materials (Supplementary Table S2). As expected, some food items had better reproducibility than others, with the best being for tomatoes (fresh, in season), lettuces, and pork lard (Supplementary Table S1). Reproducibility was lower than 0.40 for food items such as aubergines (out of season), potatoes, sweet peppers (cooked, out of season), and carrots.

**Figure 2:**
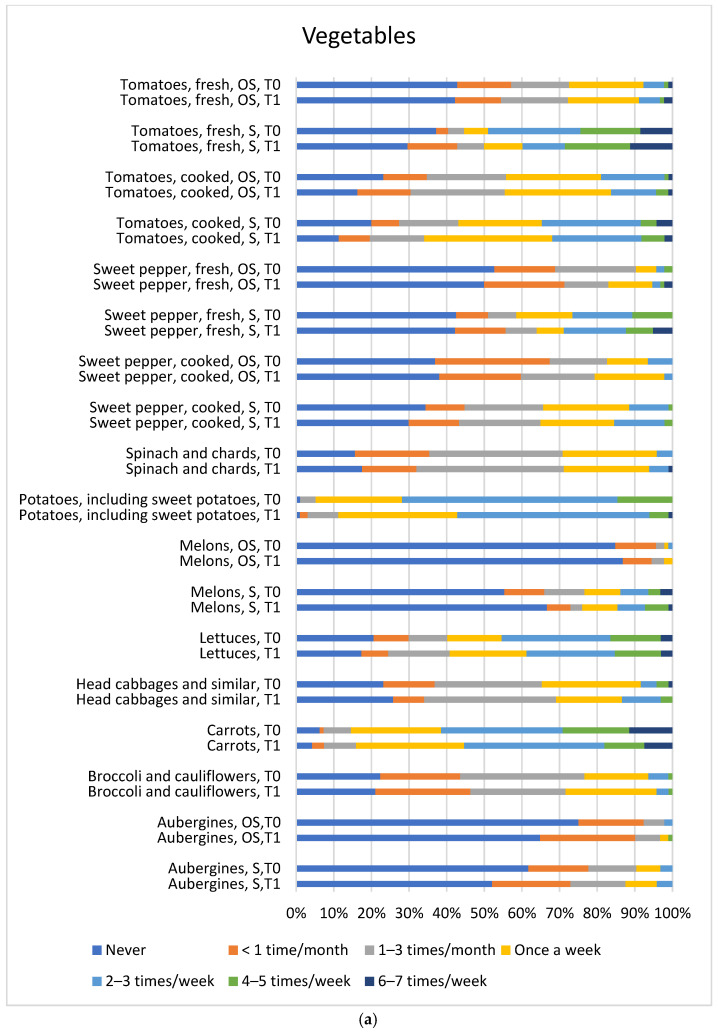

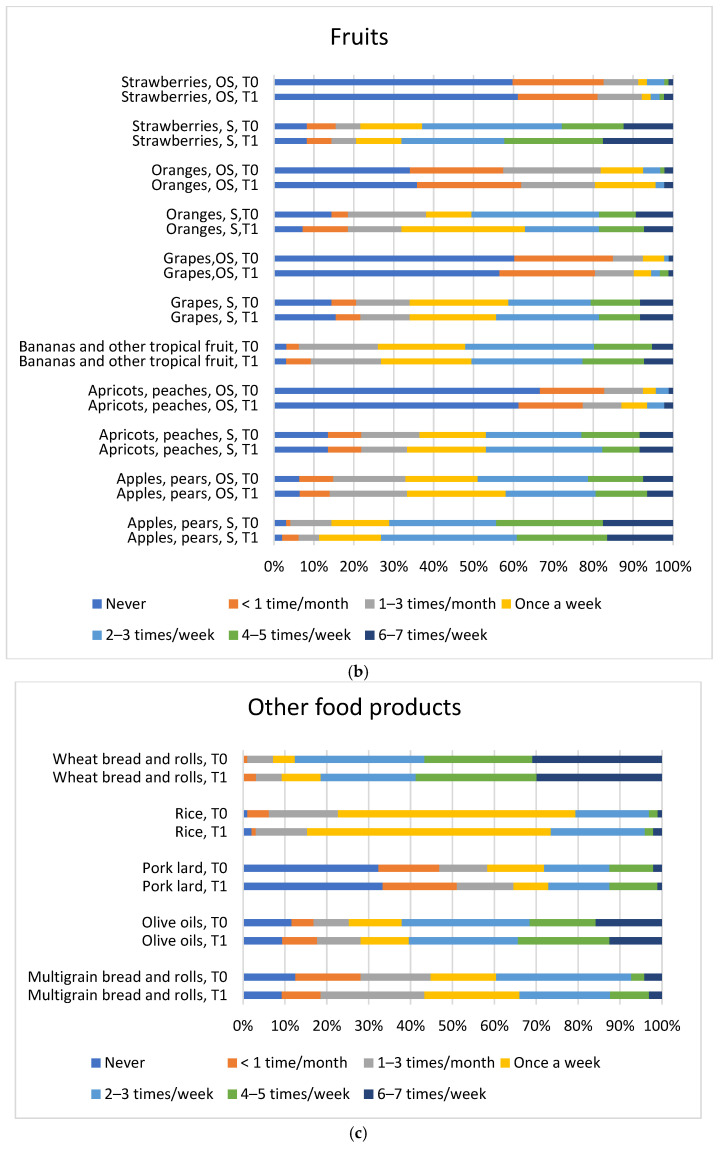
S—in season, OS—out of season (**a**) Results of the FPQ from the baseline (T0) in comparison to the repeated FPQ (T1) for vegetables from the EUCP food list. (**b**) Results of the FPQ from the baseline (T0) in comparison to the repeated FPQ (T1) for fruits from the EUCP food list. (**c**) Results of the FPQ from the baseline (T0) in comparison to the repeated FPQ (T1) for rice, wheat and multigrain bread and rolls, pork lard, and olive oils from the EUCP food list.

**Figure 3:**
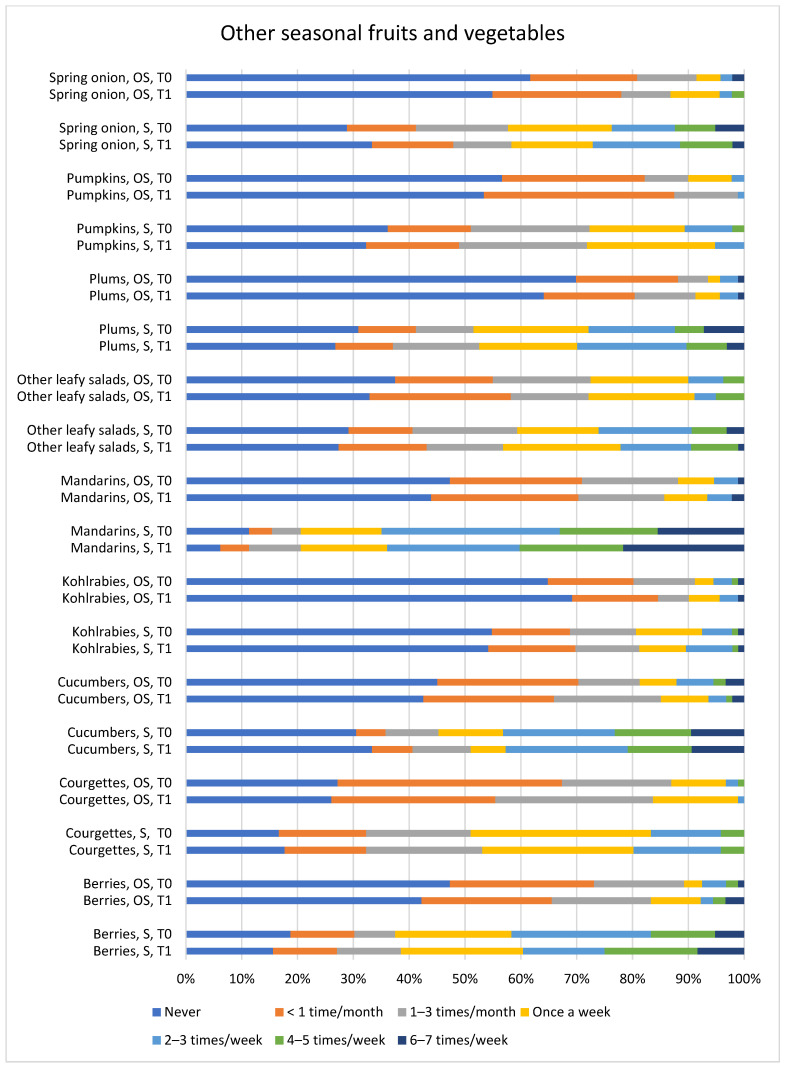
S—in season, OS—out of season. Results of the FPQ from the first round of answering (T0) in comparison to the repeated FPQ (T1) for food products that can be eaten in season and out of season.

Figure 2a–c and Figure 3 illustrate the frequency of consumption of food items with quantified PYR and OP insecticides, as reported in EUCP, alongside food items that can be eaten during both the in-season and out-of-season periods. Reported frequencies regarding fruit and fruit product consumption in season and out of season showed mostly good agreement between the two time points, with the exception of oranges: when first answering the FPQ, parents/guardians estimated a lower frequency of orange consumption during the season (at T0, 11 out of 97 vs. at T1, 30 out of 97 answered they eat an orange once a week). Similarly, estimates of vegetable and vegetable product intake in season and out of season also showed good agreement between T0 and T1, with exceptions of poor agreement for tomatoes, fresh or cooked, in season, for which the parents/guardians gave lower estimates at the first time point (at T0, 3 out of 94 vs. at T1, 13 out of 98 answered they eat a fresh tomato less than once a month; 19 out of 95 vs. 11 out of 97 answered they never eat cooked tomatoes).

Other food categories, including animal and vegetable products, fats and oils, as well as grains and grain-based products, also showed good agreement between the first and repeated FPQs (Figure 2a–c and Figure 3). Overall, from all food categories, the highest consumption frequency of wheat, bread and rolls was observed among adolescents.

Regarding seasonality, for all 20 items (Figure 2a,b and Figure 3) (aubergines, apples and pears, apricots and peaches, berries, courgettes, cucumbers, grapes, kohlrabi, lettuces, mandarins, melons, oranges, plums, pumpkin, spring onion, strawberries, sweet peppers fresh/cooked, and tomatoes fresh/cooked) there was a significant difference between consumption in season and out of season (*p* < 0.05). As expected, fruits and vegetables were eaten more in season than out of their season.

Besides FPQ, we collected data on dietary restrictions. Out of the 459 participants, only 3 did not eat meat (1%), 59 did not consume fish (13%), 23 did not consume eggs (5%), 12 did not consume milk (3%), and 22 did not consume grains (5%). There were 19 participants with food restrictions based on their health, e.g., food allergies (peanut, eggs) and/or food intolerance (gluten-free diet, lactose-free diet). Out of the 459 participants, 117 ate organic food regularly or often (25%), 267 participants sometimes ate organic food (58%), and 76 participants rarely or never ate organic food (17%). More than half of the parents/guardians grew their food at home (270 out of 459 participants, 59%), and the majority of these participants sometimes ate that food (148 out of 270 participants, 55%).

The estimated fruit intake (g/day) for adolescent boys was 262 g/day (median) with an interquartile range 176–376 g/day (mean 300 g/day).

The estimated vegetable intake (g/day) for adolescent boys was 123 g/day (median) with an interquartile range 74–190 g/day (mean 138 g/day).

## 4. Discussion

Overall, our main results show that the FPQ we designed for the estimation of pesticide exposure through dietary habits specific to adolescents exhibits good reproducibility, making it suitable for future studies investigating adverse chronic effects of pesticides on sexual development. In addition, results on adolescent dietary habits for food items that are presumed sources of PYR and/or OP residues show that the most frequently consumed foods in this age group are potatoes, carrots, lettuce, muesli, breakfast cereal, cow milk, butter, hen eggs, strawberries, oranges, grapes, apricots and peaches, apples and pears during season, and bananas.

The novelty in our study was that we focused on presumed food sources of pesticides in adolescents, especially PYR and OP, in addition to considering seasonality of fruit and vegetable consumption, and in this regard, we designed a larger food list in comparison to previous studies investigating the reproducibility of a specific FFQ design. Those food items were used to determine the frequency of consumption of PYR and OP insecticides in adolescents. For example, the Irish Food4Me study [43], on adults, tested the reproducibility of a semi-quantitative food frequency questionnaire answered twice, one month apart. Even though there was a similarity in the questionnaire layout, a direct comparison was limited by differences in methodology, specifically by the different time periods between the first and repeated questionnaires and the fact that the study used the Bland–Altman assessment due to considerations of energy intake, while we, on the other hand, employed the Cohen’s kappa method. Despite these methodological differences, both studies showed good reproducibility for the items that they both included, which confirms the robustness of our FPQ.

Similarly, a study in Greece on adults [44] measured the semi-quantitative FFQ of 14 food categories with a layout of frequency categories similar to ours. The reproducibility was administered two times within 10 to 30 days, and the same method as ours was used to evaluate the reproducibility using Cohen’s kappa statistic. Overall, Cohen’s kappa coefficient for the study by Tyrovolas et al. was below 0.50 in 13 out of 14 food categories, indicating lower reproducibility than in our study. Another Greek study [45] on adolescents, also similarly to ours, used EFSA’s recommendations, specifically the FoodEx2 programme, for designing the FPQ but deriving a shorter food list. Our study had 26 more food items on the questionnaire, the biggest difference being in the fruit category, where our study has 25 separate questions for items mostly not covered in the Greek study, in addition to having separate questions for fruits and vegetables grouped in the Greek study. In the vegetable category, for example, they grouped cucumber, pepper, pumpkin, and eggplant, which in our study were all separate questions due to them being mostly consumed in separate dishes.

Our preliminary results on dietary habits are in line with previous studies from the geographic region. From January 2018 to June 2023, Croatia conducted a national food consumption survey on adolescents and adults [28], and with it, a comparison can be made with the targeted population from our country and with neighbouring countries (Slovenia, Bosnia and Herzegovina). From the food consumption data of these three countries, we can say that Croatian and Slovenian adolescents had more similar habits compared to Bosnian adolescents. In Croatia, the highest consumption frequencies were noted for seed oils, chips/crisps, hen eggs, simple cereals which have to be reconstituted with milk or other appropriate nutritious liquids, orange juice, cattle milk, milk imitates, and tap water. For Bosnia and Herzegovina, the most frequently consumed food items were seed oils, chips/crisps, fried eggs, milk imitates, and tap water, while for Slovenian adolescents, chips/crisps, cattle milk, potatoes, and tap water. Out of the 130 food items that formed this Croatian list, 84 were the same as on the Bosnian and Slovenian food lists, 16 were the same only for the Croatian and Bosnian lists, and 30 of the food items were the same only for the Croatian and Slovenian food lists.

The current dietary guidelines for fruit and vegetable intake vary slightly among EU countries. Most guidelines recommend consuming at least five portions of a variety of fruits and vegetables each day, which corresponds to a minimum of 400 g per day. However, Spain and Greece have higher recommendations, suggesting 300 g and 360 g per day for fruits, respectively, and 450 g and 600 g per day for vegetables [46]. In Croatia, the recommended fruit and vegetable intake should be at least five portions, with a portion defined as the amount that can fit in one hand. Meanwhile, Slovenia specifies a recommendation of two to four portions of fruit per day and three to five portions of vegetables per day. Despite recommendations, the intake of fruits and vegetables among the EU adolescent population is still deficient. Additionally, older adolescents have less healthy dietary habits [47], with boys consuming fewer daily fruits and vegetables than girls [48]. A similar trend was observed in the European Childhood Obesity Initiative in Croatia in 2021/2022 on children aged eight to nine years [49], with only 3.1% of the children following the recommendation on five portions of fruits and vegetables per day. Furthermore, it was reported that girls had a slightly higher daily intake of fruits and vegetables than boys and that boys most often consume one to two servings of fruits and vegetables per day. Our findings on fruit consumption among adolescent boys show a median intake of 262 g per day (with an interquartile range of 176 to 376 g/day), which corresponds to 2.5 portions. This result may suggest an overestimation of their actual fruit intake, as it exceeds both the recommended amounts and recent data reported in the literature. Also, our preliminary results from 24-h recall data for 136 adolescent boys [50] denoted inadequate daily intake of both fruit (mean: 146 g/day) and vegetables (mean: 121 g/day). An overestimation could have happened for several reasons. Previous studies have shown a possibility of overreporting fruit intake while answering the FPQ or the FFQ due to recall bias and possible social desirability bias [21,51,52]. On the other hand, our median for estimated vegetable intake was 123 g per day (interquartile range 74–190 g/day), which is in accordance with guidelines. This could have also been overestimated. Our estimation of fruit and vegetable intake for a boy adolescent’s diet from FoodEx2 data was made based on the mean of acute consumption (g/day) and not the median, which was, in most cases, lower than the mean. Also, food data from the Croatian adolescent survey was conducted on participants aged between 10 and 18 years old, and our study was conducted on participants aged 10 to 12 years old, who could have eaten smaller portions in general. Given all this in mind, further validation of the FPQ with a 24-h recall is necessary for a more accurate estimation.

The advantage of the FPQ is that data collection with this questionnaire takes significantly less time than data collection with the FFQ, and when used in conjunction with a 24-h food diary, it is possible to obtain information on the frequency and amount of food consumed [21]. Therefore, one of the limitations is that the FPQ was not compared with a detailed dietary survey, for example, 24-h dietary recall data, as in some studies [45,53]. Another limitation was the relatively small number of participants. On the other hand, one of the strengths and novelty, as mentioned, was the design of an FPQ with specific focus on pesticide residues and considering seasonality in the questionnaire. Even though parents provide boys with meals and are more involved in their diet, it can pose a bias, as boys did not answer the FPQ by themselves. Since our FPQ was developed using data from the European Food Safety Authority (EFSA), we suggest that it be primarily used in EU countries, as pesticide usage may vary significantly in different climate areas and regulatory environments. Additionally, researchers should consider the types and quantities of pesticides utilized in their respective countries for dietary exposure assessment.

## 5. Conclusions

The proposed FPQ has shown good repeatability for food items that were identified as substantial sources of pesticides. As such, it may prove useful for future research involving PYR and OP exposure assessments. However, our findings require further validation through comparison with 24-h dietary recall data and pesticide metabolite measurements.

## Figures and Tables

**Table 1:** Characteristics of the food propensity questionnaire (FPQ) used for assessing pesticide residues intake in adolescents.

Food Groups	Questions	Seasonality
Grains and grain-based products	9	No
Vegetables and legumes (fresh, cooked, or seasoning)	38	Yes (11 questions)
Fruit and fruit-based products	25	Yes (9 questions)
Meat and meat-based products	9	No
Fish	10	No
Milk, dairy products, and milk substitutes	7	No
Eggs	1	No
Fats and oils	6	No
Sweets/sweeteners and beverages	6	No
Frequency of consumption for each food item, and each season, if applicable:	Never <1× per month 1–3× per month 1× per week 2–3× per week 4–5× per week 6–7× per week	

**Table 2:** List of food items conducted through EUCP from 2021 to 2023.

EUCP 2021 [36]	EUCP 2022 [37]	EUCP 2023 [38]
Aubergines	Head cabbage	Carrots
Broccoli	Lettuce (generic)	Cauliflowers
Fungi	Spinach	Kiwi fruits (green, red, yellow)
Sweet peppers	Tomatoes	Onions
Bananas	Apples	Oranges
Grapefruit	Peaches	Pears
Melons	Strawberries	Potatoes
Grapes	Grapes	Dried beans
Olive oil	Barley	Brown rice
Beef fat	Oat	Rye
Wheat	Cow milk	Bovine liver
Hen eggs	Pork lard	Poultry fat

## Data Availability

The data presented in this study are available on request from the corresponding author. The data are not publicly available due to privacy and ethical reasons.

## Supplementary Materials

The following supporting information can be downloaded at: https://www.mdpi.com/article/10.3390/children13030320/s1, Table S1. The list of food selected for the FPQ, with consumption data for Croatian male and Slovenian male adolescents and related inclusion criteria; Table S2. Results of the reproducibility of the food propensity questionnaire (FPQ).

## Abbreviations

The following abbreviations are used in this manuscript:

EFSA	European Food Safety Authority
EUPIC	EU coordinated control programme
FFQ	Food Frequency Questionnaire
FPQ	Food Propensity Questionnaire
OP	Organophosphate
PYR	Pyrethroids
