# Supplementary material for: Design and Reproducibility of Food Propensity Questionnaire for Characterizing Intake of Pyrethroid and Organophosphate Insecticides in Adolescents
Source: Children (Basel). 2026 Feb 25;13(3):320. doi: 10.3390/children13030320 (PMC13026074; doi:10.3390/children13030320)
Supplement: Supplementary file 1 [file children-13-00320-s001.zip › Supplementary_Table_S1_MMacan.pdf]

Table S1. The list of food selected for the FPQ, with consumption data for Croatian male and Slovenian male adolescents and related inclusion criteria.

**European Union report on pesticide residues in food for 5 years [1-4]**	**Croatian male adults [5]**	**Slovenian male adolescents [6]**	**Reason for inclusion[i]**	**Final item included in our FPQ list**
	% consumption in the category based on FoodEx2			
Apples	28.4	37.9	CRO	Apples and pears
Pears	7.8	2.1	CRO	
Apricots	0.1	0.4	EFSA	Apricots and peaches
Peaches	11.1	4.1	CRO	
Apricots processed	1.7	2.9	CRO	Fruit compote (e.g., sour cherry, apricots, pineapple)
Pineapple processed				
Plums processed	0.3	0.6	EFSA/EA	Dried fruits (e.g., raisins, apricots, figs, plums)
Table grapes processed	2.8	3.0	CRO	Jams, marmalades, and preserves
Bananas	14.7	23.9	CRO	Bananas and other tropical fruit (kiwi, pineapple, papaya, mango)
Pineapples				
Beans with pod	1.5	2.5	CRO	Beans with pods (fresh or frozen)
Brussels sprouts	NR	0.2	EFSA	Kales and Brussels sprouts
Kales	1.6	0.1	CRO	
Buckwheat and other pseudo-cereals	0.01	0.02	EFSA	Cereals other than wheat (e.g., rye, buckwheat, quinoa, barley)
Celeriacs or turnip rooted celeries	0.4	0.9	SLO, EFSA	Celeries and parsley (root, stick, leaf, fresh or cooked)
Chards or beet leaves	2.1	NR	CRO	Spinach and chard
Spinach	1.1	2.9	CRO	
Cherries (sweet)	0.3	0.7	SLO	Cherries (sweet)
Cucumbers	7.6	3.5	CRO	Cucumbers
Escaroles	0.2	0.9	EFSA	Other leafy salads (e.g., lamb's lettuce, rucola, escaroles)
Lamb's lettuces	< 0.1	0.2	EFSA	
Rucola or Roman rocket	< 0.1	0.1	EFSA	
Lettuces	4.6	12.4	CRO	Lettuces
Gherkins	3.2	2.5	CRO	Gherkins, sour pepper
Herbal infusions, not specific (dried)	13.8	29.2	CRO	Herbal infusions, not specific (dried)

European Union report on pesticide residues in food for 5 years [1-4]	Croatian male adults [5]	Slovenian male adolescents [6]	Reason for inclusion[i]	Final item included in our FPQ list
	% consumption in the category based on FoodEx2			
Kohlrabies	NR	0.5	EFSA	Kohlrabies
Lemons	0.5	0.1	EFSA	Lemons (juice, zest)
Mandarins	7.4	7.4	CRO	Mandarins
Melons	18.7	7.0	CRO	Melons
Oranges	0.8	5.9	EFSA	Oranges
Pumpkin seeds processed (oil)				Other types of vegetable oils and fats (e.g., vegetable, rapeseed, pumpkin, sesame, coconut)
Raspberries	0.2	0.0	EFSA	Other berries (e.g., raspberries, blueberries, blackberries)
Rice and processed rice	1.9	2.6	CRO	Rice
Strawberries	0.04	0.9	EFSA	Strawberries
Sweet peppers/ bell peppers	7.1	5.7	CRO	Sweet peppers/ bell peppers, fresh
				Sweet peppers/ bell peppers, thermally processed
Table grapes	17.5	1.7	CRO	Table grapes
Teas	3.9	15.1	CRO	Teas (green, black, white)
Tomatoes	22.8	15.0	CRO	Tomatoes, fresh
				Tomatoes, cooked
Tomatoes processed	2.6	4.2	CRO	Tomatoes, canned (pureed, concentrate)
Turnips	< 0.1	2.7	EA	Beetroot and turnips, including sour
Cultivated fungi	2.1	3.8	CRO	(Not included due to low consumption)
Wild fungi				
Figs	1.0	0	CRO	
Pomegranates	1.0	0.5	EFSA	
	3.2	2.9	CRO	Plums
	13.8	26.7	CRO, SLO	Nuts (e.g., walnuts, hazelnuts, almonds) and peanuts
	99.9	100.0	CRO	Potatoes, including sweet potato
	0.2/0.5	2.5/1.9	EFSA, EA	Broccoli and cauliflower

**European Union report on pesticide residues in food for 5 years [1-4]**	**Croatian male adults [5]**	**Slovenian male adolescents [6]**	**Reason for inclusion[i]**	**Final item included in our FPQ list**
	% consumption in the category based on FoodEx2			
	1.3	3.8	CRO	Fermented vegetables (sauerkraut)
	7.6	3.5	CRO	Courgettes
	0.7	NR	EA	Aubergine
	3.4	7.4	CRO	Carrot, fresh or cooked
	NR	0.3	EA	Spring onion
	0.2	1.2	EFSA	Leek
	18.7	7.0	CRO	Pumpkins
				Asparagus
	77.8	59.4	CRO, EA	Legumes (e.g., beans, peas, lentils) and legume products (e.g., tofu, hummus, lentil chips)

References:

1. European Food Safety Authority. The 2015 European Union report on pesticide residues in food. EFSA Journal 2017; 15(4):4791, 134 pp. https://doi.org/10.2903/j.efsa.2017.4791.
2. European Food Safety Authority. The 2016 European Union report on pesticide residues in food. EFSA Journal 2018;16(7):5348, 139 pp. https://doi.org/10.2903/j.efsa.2018.5348.
3. European Food Safety Authority. Scientific report on the 2017 European Union report on pesticide residues in food. EFSA Journal 2019;17(6):5743, 152 pp. https://doi.org/10.2903/j.efsa.2019.5743.
4. European Food Safety Authority; Medina-Pastor, P.; Triacchini, G. The 2018 European Union report on pesticide residues in food. EFSA Journal 2020, 18(4):6057, 103 pp. https://doi.org/10.2903/j.efsa.2020.6057.
5. Sokolić, D.; Jurković, M.; Bašić, S.; Mikec, D., Ileš, D.; Libl Vargović L.; Srdarević M. Croatian National Food Consumption Survey on Adult Population. EFSA supporting publication 2017, EN-1297. 18 pp. https://doi.org/10.2903/sp.efsa.2017.EN-1297.
6. Gregorič, M.; Blaznik, U.; Delfar, N.; Zaletel, M.; Lavtar, D.; Koroušić Seljak, B.; Golja, P.; Zdešar Kotnik, K.; Pravst, I.; Fidler Mis, N.; et al. Slovenian national food consumption survey in adolescents, adults and elderly. EFSA supporting publication 2019, EN-1729. 28 pp. https://doi.org/10.2903/sp.efsa.2019.EN-1729.

---

[i] **CRO**: food item added because it is among foods that comprise at least 95% of the total diet weight for Croatian male adults; **SLO**: food item added since it is typical for Slovenian male adolescents, compared to Slovenian male adults, and it is among foods that comprise at least 95 % of the total diet weight for Slovenian male adolescents; **EFSA**: food item not among foods that comprise at least 95 % of the total diet weight for Croatian male adults or Slovenian male adolescents, but it is an important contributor to dietary exposure to pesticides (based on EFSA reports); **EA**: expert's advice, i.e. food item added based on advice provided by the national expert (in the field of pesticide exposure and human health risk assessment or in the field of nutrition).
