# Supplementary material for: Design and Reproducibility of Food Propensity Questionnaire for Characterizing Intake of Pyrethroid and Organophosphate Insecticides in Adolescents
Source: Children (Basel). 2026 Feb 25;13(3):320. doi: 10.3390/children13030320 (PMC13026074; doi:10.3390/children13030320)
Supplement: Supplementary file 1 [file children-13-00320-s001.zip › Supplementary_Table_S2_MMacan.pdf]

Table S2. Results of the reproducibility of the food propensity questionnaire (FPQ).

Food item	Kappa [95 % CI]	Spearman rho	p-value
**Grain and grain-based products**			
Wheat bread and rolls	0.56 [0.39, 0.72]	0.53	< 0.01
Multigrain bread and rolls	0.54 [0.39, 0.70]	0.57	< 0.01
Cereal flakes	0.59 [0.45, 0.73]	0.63	< 0.01
Pasta and pasta-like products	0.54 [0.35, 0.73]	0.51	< 0.01
Cakes and biscuits	0.53 [0.39, 0.66]	0.53	< 0.01
Rice	0.52 [0.29, 0.73]	0.42	< 0.01
Cereal grains other than wheat	0.65 [0.48, 0.81]	0.66	< 0.01
Sweet corn, including canned	0.49 [0.31, 0.69]	0.49	< 0.01
Crackers, sticks (salty), popcorn, etc.	0.51 [0.31, 0.72]	0.55	< 0.01
**Vegetables, legumes, and vegetable products**			
Potatoes and sweet potatoes	0.29 [0.07, 0.52]	0.39	< 0.01
Lettuces (generic)	0.85 [0.77, 0.92]	0.83	< 0.01
Lettuces and salad plants – in season (e.g., Lamb's lettuce, radicchio)	0.77 [0.66, 0.87]	0.77	< 0.01
Lettuces and salad plants – out of season (e.g., Lamb's lettuce, radicchio)	0.58 [0.38, 0.79]	0.56	< 0.01
Spinach-type leaves (spinach and chard)	0.72 [0.63, 0.82]	0.72	< 0.01
Broccoli and cauliflowers	0.69 [0.57, 0.83]	0.68	< 0.01
Kales and Brussels sprouts	0.54 [0.38, 0.70]	0.54	< 0.01
Head cabbages	0.68 [0.57, 0.78]	0.69	< 0.01
Sauerkraut	0.60 [0.42, 0.79]	0.68	< 0.01
Tomatoes, fresh, in season (May to October)	0.86 [0.81, 0.92]	0.86	< 0.01
Tomatoes, fresh, out of season	0.68 [0.55, 0.81]	0.69	< 0.01
Tomatoes, cooked, in season (May to October)	0.54 [0.38, 0.69]	0.50	< 0.01
Tomatoes, cooked, out of season	0.45 [0.25, 0.65]	0.41	< 0.01
Preserved concentrated tomatoes	0.29 [0.03, 0.57]	0.35	< 0.01
Peppers, fresh, in season (May to September)	0.70 [0.57, 0.84]	0.70	< 0.01
Peppers, fresh, out of season	0.45 [0.21, 0.69]	0.54	< 0.01
Peppers, cooked, in season (May to September)	0.41 [0.21, 0.60]	0.42	< 0.01
Peppers, cooked, out of season	0.37 [0.18, 0.56]	0.44	< 0.01
Courgettes, in season (July, August)	0.61 [0.44, 0.78]	0.61	< 0.01

Food item	Kappa [95 % CI]	Spearman rho	p-value
Courgettes, out of season	0.47 [0.31, 0.64]	0.48	< 0.01
Cucumbers, in season (April to July)	0.77 [0.67, 0.87]	0.77	< 0.01
Cucumbers, out of season	0.68 [0.53, 0.83]	0.61	< 0.01
Gherkins	0.76 [0.65, 0.88]	0.76	< 0.01
Beetroots and turnips, including sour beetroots and turnips	0.68 [0.53, 0.82]	0.69	< 0.01
Aubergines, in season (June to September)	0.63 [0.45, 0.81]	0.62	< 0.01
Aubergines, out of season	0.28 [0.04, 0.51]	0.43	< 0.01
Carrots	0.39 [0.16, 0.62]	0.43	< 0.01
Celeriacs and parsley roots	0.49 [0.32, 0.66]	0.51	< 0.01
Kohlrabies, in season (May to October)	0.56 [0.37, 0.74]	0.52	< 0.01
Kohlrabies, out of season	0.39 [0.18, 0.61]	0.51	< 0.01
Spring onions, in season (April to June)	0.69 [0.55, 0.82]	0.72	< 0.01
Spring onions, out of season	0.51 [0.27, 0.75]	0.54	< 0.01
Leeks	0.62 [0.42, 0.81]	0.64	< 0.01
Pumpkins, in season (July to October)	0.59 [0.43, 0.75]	0.62	< 0.01
Pumpkins, out of season	0.36 [0.18, 0.54]	0.46	< 0.01
Asparagus, in season (April to June)	0.69 [0.54, 0.83]	0.76	< 0.01
Peas with pods, fresh or frozen	0.73 [0.63, 0.82]	0.73	< 0.01
Legumes (e.g., beans, peas without pods, lentils, chickpeas) and legume products (e.g., tofu, hummus)	0.59 [0.44, 0.75]	0.55	< 0.01
**Fruit, nuts, and fruit products**			
Oranges, in season (November to March)	0.67 [0.55, 0.79]	0.64	< 0.01
Oranges, out of season	0.58 [0.42, 0.75]	0.51	< 0.01
Mandarins, in season (November to January)	0.68 [0.56, 0.81]	0.65	< 0.01
Mandarins, out of season	0.71 [0.57, 0.84]	0.71	< 0.01
Lemons (juice, zest)	0.52 [0.33, 0.71]	0.52	< 0.01
Common banana	0.68 [0.55, 0.80]	0.66	< 0.01
Cherries (sweet), in season (May to July)	0.81 [0.71, 0.89]	0.79	< 0.01
Strawberries, in season (May, June)	0.72 [0.59, 0.85]	0.66	< 0.01
Strawberries, out of season	0.59 [0.36, 0.84]	0.59	< 0.01
Berries (e.g., raspberries, blackberries, blueberries), in season (June to August)	0.72 [0.59, 0.86]	0.74	< 0.01
Berries (e.g., raspberries, blackberries, blueberries), out of season	0.58 [0.34, 0.82]	0.55	< 0.01

Food item	Kappa [95 % CI]	Spearman rho	p-value
Apples and pears, in season (June to October)	0.77 [0.68, 0.87]	0.71	< 0.01
Apples and pears, out of season	0.65 [0.48, 0.81]	0.69	< 0.01
Plums, in season (August, September)	0.62 [0.49, 0.75]	0.62	< 0.01
Plums, out of season	0.58 [0.33, 0.84]	0.58	< 0.01
Apricots and common peaches, in season (June to August)	0.69 [0.58, 0.79]	0.69	< 0.01
Apricots and common peaches, out of season	0.59 [0.42, 0.76]	0.55	< 0.01
Table grapes, in season (July to September)	0.69 [0.55, 0.82]	0.65	< 0.01
Table grapes, out of season	0.63 [0.47, 0.79]	0.58	< 0.01
Melons, in season (July, August)	0.69 [0.56, 0.83]	0.67	< 0.01
Melons, out of season	0.49 [0.17, 0.82]	0.23	< 0.01
Dried fruit (e.g., raisins, dried figs, dried apricots, dried plums)	0.59 [0.42, 0.76]	0.61	< 0.01
Tree nuts (e.g., cashew nuts, almond sweet, hazelnuts, walnuts) and peanuts	0.61 [0.45, 0.76]	0.56	< 0.01
Fruit compote (e.g., apricot, sour cherry)	0.53 [0.35, 0.71]	0.53	< 0.01
Fruit jelly, jam of fruits, marmalade	0.67 [0.54, 0.81]	0.68	< 0.01
**Meat and meat products**			
Beef and calf fresh meat	0.63 [0.48, 0.79]	0.59	< 0.01
Pig fresh meat	0.64 [0.49, 0.79]	0.57	< 0.01
Lamb, sheep, and goat fresh meat	0.62 [0.43, 0.79]	0.67	< 0.01
Birds meat (chicken, duck, turkey)	0.58 [0.36, 0.80]	0.47	< 0.01
Processed whole meat products (e.g., ham), sausages (e.g., bologna-type salami, salami-type sausages), and meat spread	0.45 [0.22, 0.67]	0.47	< 0.01
Frankfurter sausages	0.48 [0.27, 0.69]	0.51	< 0.01
Bacon or pancetta	0.61 [0.48, 0.74]	0.62	< 0.01
Mammals or birds slaughtering products (e.g., liver, kidney)	0.64 [0.46, 0.82]	0.64	< 0.01
Liver-based spreadable textured specialities (e.g., chicken, pork)	0.68 [0.54, 0.82]	0.65	< 0.01
**Fish and seafood**			
Small marine fish (e.g., sardines, anchovies, mackerel), fresh or canned	0.53 [0.39, 0.67]	0.56	< 0.01
Large marine fish (e.g., bonito, greater amberjack, swordfish)	0.44 [0.24, 0.65]	0.55	< 0.01

Food item	Kappa [95 % CI]	Spearman rho	p-value
Tuna	0.56 [0.36, 0.75]	0.54	< 0.01
Canned tunas	0.51 [0.34, 0.68]	0.59	< 0.01
Gilthead bream, hake, seabass, scorpion fish, wild-caught	0.44 [0.25, 0.64]	0.42	< 0.01
Gilthead bream, hake, cultivation	0.69 [0.56, 0.82]	0.72	< 0.01
Freshwater fish (e.g., trout, carp, pike)	0.54 [0.32, 0.77]	0.46	< 0.01
Processed or preserved seafood (e.g., fish fingers, breaded fish)	0.49 [0.35, 0.64]	0.50	< 0.01
Shrimps and prawns	0.71 [0.59, 0.82]	0.72	< 0.01
Squids, octopus, cuttlefish	0.71 [0.59, 0.82]	0.74	< 0.01
**Milk and dairy products**			
Cow milk, whole	0.62 [0.44, 0.79]	0.53	< 0.01
Soya drink, almond drink, oat drink, etc.	0.62 [0.44, 0.81]	0.73	< 0.01
Goat milk	0.54 [0.40, 1.00]	0.39	< 0.01
Yoghurt (cow milk, plain), including kefir, acidophilus milk	0.61 [0.48, 0.74]	0.62	< 0.01
Cottage cheese	0.66 [0.53, 0.79]	0.66	< 0.01
Semi-hard and hard cheese	0.60 [0.44, 0.76]	0.55	< 0.01
Ice cream, milk-based	0.39 [0.24, 0.54]	0.48	< 0.01
**Eggs**			
Hen eggs	0.67 [0.52, 0.82]	0.66	< 0.01
**Animal and vegetable fats and oils**			
Butter	0.64 [0.49, 0.79]	0.66	< 0.01
Traditional margarine	0.62 [0.44, 0.80]	0.67	< 0.01
Sunflower seed oil, edible	0.60 [0.42, 0.79]	0.58	< 0.01
Olive oil	0.73 [0.62, 0.84]	0.75	< 0.01
Vegetable fats and oils, edible (e.g., rape seed oil, linseed oil, hemp seed oil)	0.39 [0.15, 0.65]	0.37	< 0.01
Pork lard	0.85 [0.77, 0.92]	0.84	< 0.01
**Sugar and similar, tea, infusions, and water-based beverages**			
Honey	0.62 [0.45, 0.79]	0.60	< 0.01
Fruit juice concentrate, fruit juices and nectars	0.47 [0.31, 0.63]	0.49	< 0.01
Fruit juices (100 % from named source)	0.47 [0.29, 0.66]	0.48	< 0.01
Tea beverages	0.49 [0.31, 0.66]	0.55	< 0.01
Herbal and other non-tea infusions	0.66 [0.54, 0.77]	0.67	< 0.01
Soft drinks (e.g., cola beverages, fruit soft drink orange)	0.69 [0.59, 0.81]	0.72	< 0.01
